# Protecting Public Health: Stories of Adaptation from Communities Across the Globe

**DOI:** 10.5334/aogh.4976

**Published:** 2025-12-19

**Authors:** Praveen Kumar, Stella Hartinger, Sokhna Thiam, Amit Mistry, Jenna R. Durham

**Affiliations:** 1 Associate Professor, Boston College School of Social Work, Chestnut Hill, MA, USA; 2 Associate Professor, Co‑director – Centro Latino Americano de Excelencia en Cambio Climático y Salud, Universidad Peruana Cayetano Heredia, Lima, Perú; 3 Associate Research Scientist, West Africa Regional Office (WARO), African Population and Health Research Center (APHRC), Dakar, Senegal; 4 Independent Researcher, Bethesda, Maryland, USA; 5 Science Policy Analyst, N4 Solutions, LLC, Columbus, Ohio, USA

## Background

Global climate change has exacerbated health risks and substantially contributed to adverse health outcomes that vary both temporally and spatially [1]. Existing evidence confirms an unprecedented rise in the incidence of climate‑led stressors, including, but not limited to, increased ambient temperatures, erratic precipitation, extreme weather events, sea level rise, wildfires, and desertification [2]. The climate stressors have exacerbated exposure to conditions such as high heat stress, reduced air quality, disruptions to food and water systems, migration and internal displacement, resource conflicts, and changes in infectious agents [1]. This has led to worsening public health outcomes such as premature mortality, heat‑related illnesses, increased vector‑borne diseases, malnutrition, and mental health consequences [3]. Health inequities, primarily a function of social, economic, behavioral, and institutional sub‑systems, have further deteriorated due to climate change [4]. There exists a broad consensus that the consequences for health are greatest in low‑ and middle‑income countries (LMICs). However, not enough attention has been paid to address the climate‑induced health consequences in the LMICs. Moreover, despite the successes achieved by the global community in mitigating emissions, climate shifts continue and are expected to worsen in multiple geographies for decades to come. This calls for urgent attention to identify adaptation strategies that can respond to public health risks due to climate change in LMICs.

Adaptation is broadly defined by the IPCC (2014) as the process of adjustment to actual and potential climate‑led impacts [5]. Examples of adaptation strategies include heat‑resistant crops, behavioral changes, effective climate communication and awareness building, green infrastructure, wetland restoration, coastal land preservation, and microfinancing [2, 6]. While adaptation may not reduce the larger climate‑led stressors, it minimizes the exposure pathways, thereby abating the adverse consequences of climate change on human health.

This special collection is intended to address the current gaps in the literature regarding the impact of climate change adaptation on health, which are, for instance, (1) inadequate information on effective adaptation strategies that impact health, (2) innovative research approaches to better understand the impact of climate adaptation on health, (3) theoretical and methodological challenges to study climate adaptation and health, and (4) relatively low contributions from researchers and practitioners from the LMICs. The primary objective of the collection is to contribute to building a solutions‑oriented science focused on the health threats posed by global climate change and to increase the visibility of adaptation research already being conducted at the local level in many low‑resource settings around the world.

### Selection of case studies

To address the gaps in research on climate adaptation strategies in LMICs, the Fogarty International Center (FIC) of the U.S. National Institutes of Health (NIH), in partnership with the NIH Climate Change and Health Initiative, invited submissions from the global health community between August and November 2023. Submissions included proposals for case studies that outlined an adaptation strategy used in an LMIC setting and a proposed analysis of the impacts of the adaptation strategy on health. More than 150 proposals were received, and after an initial screening based on relevance to the call, 133 proposals were selected for further review. These proposals came from 40 different countries and were submitted by academics, NGOs, and private organizations. A Steering Committee of diverse global experts reviewed the proposals based on the following criteria: scientific and public health relevance, scientific approach, and diversity in terms of study location, submitting authors, health outcomes, climate stressors involved, and type of adaptation strategy. After the review and scoring process, a total of 15 proposals, one each from 15 different countries, were selected by the Steering Committee to be developed into full case study articles. This collection includes 14 completed case studies as one team was unable to complete its proposed case study. This editorial provides an overview of the collection and highlights several themes that cut across the collection.

### Overview of selected case studies

The 14 case studies represent a range of climate adaptation strategies, health outcomes, environmental stressors, and populations of focus. Five case studies are from Africa (Chad, Ethiopia, Kenya, Madagascar, and Nigeria), four are from Asia (Federated States of Micronesia [FSM], India, Pakistan, and Thailand), and five are from Latin America and the Caribbean (Brazil, Guatemala, Mexico, Nicaragua, and multiple small island nations in the Caribbean). The collection has focused on diverse population categories, including pastoralists, pregnant women, coastal residents, agricultural workers, hospital patients, older adults, children, and state officials. The case studies analyzed climate stressors, ranging from drought and extreme weather events to excessive rain and high heat stress. For instance, cases from Madagascar, Kenya, Thailand, and the Caribbean islands have primarily focused on high precipitation and flooding, whereas Ethiopia, Brazil, and Mexico have focused on drought.

The case studies focused on diverse public health issues as outcomes of climate stressors. For instance, cases from three countries (Pakistan, Nicaragua, and Guatemala) discussed the heat‑related illnesses, two cases (Ethiopia and India) on vector‑borne diseases, two cases on nutrition (Chad and FSM), and one case on mental health (Thailand), and one on maternal and child health (Nigeria). The remaining five cases (Madagascar, Kenya, Brazil, the Caribbean islands, and Mexico) focused on multiple health outcomes.

The case studies demonstrate how adaptation strategies have addressed detrimental health outcomes due to climate stressors. The collection of adaptation strategies largely skews toward deploying community engagement as a primary approach to address negative health outcomes. Seven cases (Chad, Madagascar, Nigeria, Pakistan, FSM, Guatemala, and the Caribbean islands) have focused on community engagement. There is one case study each on infrastructure design (Nicaragua) and surveillance and early warning systems (Ethiopia). The remaining five cases (Kenya, India, Thailand, Brazil, and Mexico) focused on a group of complementary adaptation strategies, including community engagement, infrastructure design, and/or land‑use planning. Table 1 summarizes the distribution of climate stressors, health outcomes, and adaptation strategies.

**Table 1:** Climate stressors, health outcomes, and adaptation strategies across country case studies.

CASE STUDY COUNTRY	CLIMATE STRESSOR	HEALTH OUTCOMES	ADAPTATION STRATEGY
***Africa***			
Chad	Multiple	Nutrition	Community engagement and capacity building
Ethiopia	Precipitation extremes	Vector‑borne diseases	Surveillance and early warning systems
Kenya	Air quality	Multiple	Multiple
Madagascar	Extreme weather events	Multiple	Community engagement and capacity building
Nigeria	Precipitation extremes	Maternal and child health	Community engagement and capacity building
***Asia***			
Federated States of Micronesia	Multiple	Nutrition	Community engagement and capacity building
India	Extreme weather events	Vector‑borne diseases	Multiple
Pakistan	Increased temperatures	Heat‑related illnesses	Community engagement and capacity building
Thailand	Precipitation extremes	Mental health	Flood management
***Latin America and The Caribbean***			
Brazil	Precipitation extremes	Multiple	Multiple
Guatemala	Increased temperatures	Heat‑related illnesses	Community engagement and capacity building
Mexico	Multiple	Multiple	Multiple
Caribbean Small Island Nations	Multiple	Multiple	Community engagement and capacity building
Nicaragua	Increased temperatures	Heat‑related illnesses	Infrastructure design and weatherization

*Note:* The term “multiple” denotes that the case study focuses on more than one climate stressor, health outcome, and/or adaptation strategy.

## Discussion

The selected 14 case studies represent a snapshot of multiple climate adaptation strategies being deployed in LMICs and their impact on public health. These case studies provide the best practices on adaptation strategies that have an effect on health outcomes. The collection provides a few insights that are worth highlighting, particularly to underscore promising areas for future research and funding, as well as existing gaps. They are briefly listed below.

*First*, when it comes to adaptation strategies, there is no one‑size‑fits‑all solution. Deliberate support for research conducted by and within LMICs is essential, as local context and knowledge of adaptation strategies and health outcomes yield better results and more locally relevant solutions. Adaptation strategies must be context‑specific, tailored to the unique needs of each country and community, while building upon existing research and successful interventions. For instance, promoting the cultivation of locally grown, climate‑resistant crops and food preservation techniques practiced in local communities, as discussed in the FSM case study, can reduce food insecurity caused by extreme precipitation challenges and has a greater reception among community members. Seemingly unique and successful adaptation interventions require a thorough understanding of local contextual factors and necessary modifications for successful implementation in diverse geographical settings.

*Second*, in addition to positive health outcomes, a few case studies also focused on maladaptation (cases from Kenya, the FSM, and Thailand). These additions are crucial for the continued research and implementation of climate adaptation measures. It is not enough to know what works or what does not, but also to understand components of adaptation strategies that could exacerbate the public health situation.

*Third*, all 14 cases demonstrate the need for cross‑sectoral collaboration for the successful deployment of adaptation strategies. Partnerships with local communities, governments, local non‑profits, and research institutions unlock the necessary skills and resources needed for a successful implementation of climate adaptation strategies [7, 8]. The cases from Kenya, Thailand, India, the FSM, and Pakistan demonstrate that the detrimental impacts of climate change are projected to put further strains on the already fragile healthcare institutions and services in LMICs. Lack of participatory adaptation strategies will further exacerbate these challenges.

*Fourth*, the collection only has one study that focuses on mental health (Thailand) and one study on maternal and child health (MCH) outcomes (Nigeria) in the context of climate change adaptation. This suggests that there is still a need for more research, given the outsized impact on these health areas. There has been a clarion call for increased attention to these two health outcomes [8]. Climate stressors have been shown to affect mental health outcomes, including increased anxiety, depression, stress, and post‑traumatic stress disorder. Studies on physical health outcomes, however, still vastly outnumber mental health in the climate and health literature [2, 8].

*Fifth*, the Thailand case study examines how flood adaptive strategies, including infrastructure management and community engagement, impact the cognitive health of middle‑aged and older adult populations. The study underscores the importance of tailoring climate adaptation program components specifically for older adults, particularly in aging societies.

*Sixth*, cases from India and Kenya highlight the need to develop locally relevant measures for evaluating health outcomes and adaptation interventions. For instance, implementation frameworks and mental health measures for depression, anxiety, stress, and PTSD used to evaluate these adaptation interventions are mostly developed based on studies in the US and other high‑income countries. These frameworks struggle with local disconnects when deployed in LMICs.

## Conclusion

Communities in LMICs face a disproportionate climate‑related health burden, compounded by the lack of preparedness of their health systems and inadequate policy frameworks. Unfortunately, current literature on climate change adaptation often lacks a dedicated focus on public health concerns, creating a critical gap in actionable strategies for these vulnerable populations. For instance, there is a paucity of population health research, including longitudinal studies, to examine the chronic health impacts of climate change. Lack of targeted funding on climate change and health further exacerbates the challenge. It is incumbent on scholars and practitioners to develop and share evidence‑based adaptation strategies that could have a positive impact on health outcomes. This collection aims to provide a global platform for scholars and practitioners, particularly from the LMICs, to share their learnings with the broader community. This is crucial as the LMICs grapple with the most severe health impacts due to climate change; however, their perspectives are underrepresented. We hope that this collection will foster cross‑fertilization of ideas that will help accelerate adaptation solutions for improving health outcomes at the international and local levels.
